# Unexplained Acute Homonymous Hemianopia as a Presentation of Creutzfeldt-Jakob Disease

**DOI:** 10.7759/cureus.101513

**Published:** 2026-01-14

**Authors:** Omua Esezoobo, David Gosal, KeiraAnnie Markey

**Affiliations:** 1 Department of Neurology, Manchester Centre for Clinical Neurosciences, Northern Care Alliance NHS Foundation Trust, Manchester, GBR; 2 Faculty of Health and Medicine, Lancaster University, Lancaster Medical School, Bailrigg, GBR

**Keywords:** creutzfeldt–jakob disease, functional neurological disorder, heidenhain variant, homonymous hemianopia, prion diseases, rt-quic, stroke mimic

## Abstract

Creutzfeldt-Jakob disease (CJD) is the most common prion disorder that affects humans. The Heidenhain variant (HvCJD) of sporadic CJD (sCJD) is characterized by an array of often bizarre visual symptoms, which may precede progressive fatal neurodegeneration. Here, we describe a patient who presented with left homonymous hemianopia following a fall. Initial MRI and workup for vascular, autoimmune, infectious, paraneoplastic, or metabolic causes were unremarkable. Ophthalmological assessments suggested unexplained visual field loss with a possible functional overlay due to variability on examination. Over the following month, her condition deteriorated with progressive impaired awareness, involuntary movements, and finally akinetic mutism. Serial electroencephalography (EEG) demonstrated abnormalities consistent with significant encephalopathy with the development of periodic discharges. Further magnetic resonance imaging (MRI) showed parieto-occipital cortical ribboning. Altogether, the findings were consistent with a diagnosis of prion disease, more specifically, the Heidenhain or posterior variant of the condition. She died within six weeks of presentation. This report highlights the diagnostic challenges posed by HvCJD in patients with unexplained visual field loss, which can mimic a posterior circulation stroke or a functional neurological disorder. A normal MRI scan does not exclude CJD, especially in the early stages of the disease. Serial MRI and EEGs are needed in patients who present with unexplained and rapidly progressive encephalopathy with associated neurological symptoms. Early CSF analysis, including real-time quaking-induced conversion (RT-QuIC), should be considered in similar cases.

## Introduction

Prion diseases are a group of rare transmissible neurodegenerative disorders, with Creutzfeldt-Jakob disease (CJD) being the most common in humans [1]. Most cases of CJD occur sporadically with a global incidence of one to two per million per year [1]. The Heidenhain variant (HvCJD) is a rare manifestation of sporadic CJD (sCJD), only accounting for an average of <5% of cases [2]. It is characterized by early isolated visual symptoms, which reflect the predilection of prions for the occipital cortex [2]. Due to its rapid progression and low incidence, HvCJD is seldom initially suspected by physicians, leading to a delay in performing useful diagnostic tests, such as a cerebrospinal fluid (CSF) analysis [3]. Although real-time quaking-induced conversion (RT-QuIC), tau and 14-3-3 protein testing provide high sensitivity for prion disease, a lumbar puncture is not always pursued initially when other more likely diagnoses, such as stroke, are suspected.

The sensitivity of magnetic resonance imaging (MRI) with diffusion-weighted imaging (DWI) and corresponding apparent diffusion coefficient (ADC) in CJD diagnosis exceeds 90%, with fluid attenuated inversion recovery (FLAIR) providing supportive findings [4]. Typical findings of asymmetric cortical hyperintensities (cortical ribboning), basal ganglia involvement, particularly the caudate nucleus and putamen, and increased thalamic signal (‘pulvinar sign’) are found, which correspond to the area of spongiform changes, the pathognomonic histopathological change in CJD [4]. However, early on in Heidenhain presentations, MRI may be unremarkable [5].

We report a case of HvCJD with unexplained sudden visual loss, highlighting the importance of considering other differentials, including prion disease, despite early normal imaging.

## Case presentation

A 77-year-old woman with a background of type 2 diabetes mellitus was urgently referred to the neurology clinic following a backward fall at home, which occurred after a sudden episode of premorbid trigeminal neuralgia two weeks earlier. Since the fall, she reported visual disturbance, including blurred vision, diplopia, and wavy lines. She had previously been reviewed for 'forgetfulness' several months earlier, along with progressively worsening balance, which had been attributed to residual deficits from a previous presumed stroke. The patient described a change in colour vision with an intense perception of blue, intermittent diplopia, and poor coordination. Neurological examination revealed a left homonymous hemianopia, subtle bidirectional gaze-evoked nystagmus, normal unrestricted eye movements, and an ataxic gait.

**Figure 1:**
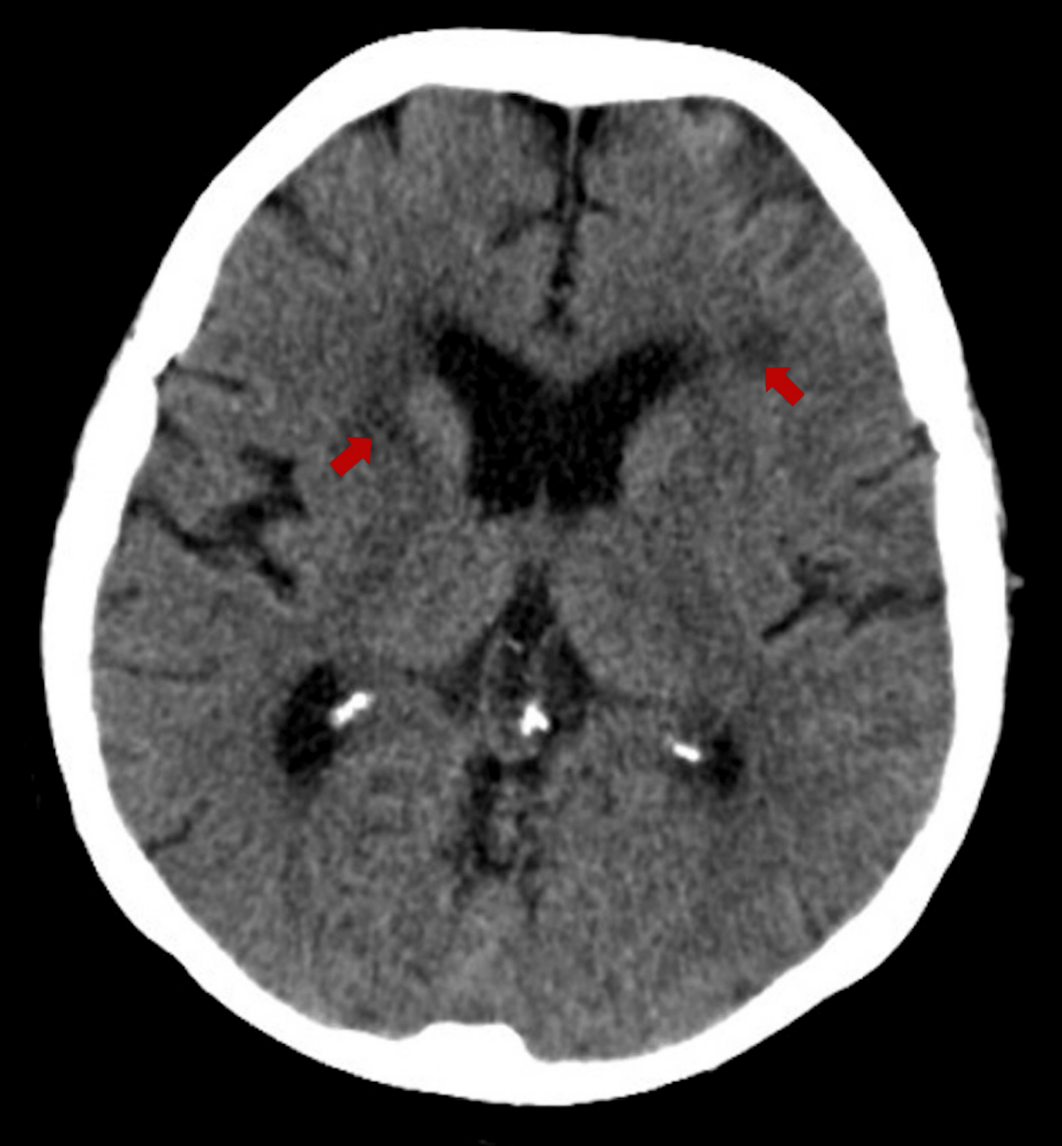
CT head without contrast, performed in the emergency department. Findings shown: Periventricular microvascular ischaemic changes with no acute haemorrhage or large artery territory infarction.

**Figure 2:**
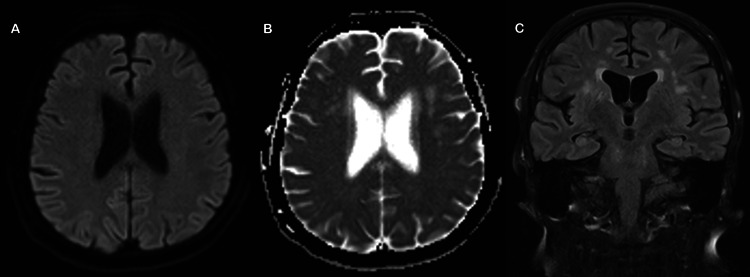
Initial MRI performed in the emergency department. Findings shown: A) DWI axial demonstrates no acute infarction; B) apparent diffusion coefficient (ADC) axial shows chronic ischaemic changes; and C) fluid-attenuated inversion recovery (FLAIR) coronal shows similar ischaemic changes.

Following the neurology outpatient review, the patient was sent directly to the emergency department on suspicion of a posterior circulation stroke. Computed tomography (CT) showed chronic microvascular disease and old lacunar infarcts but no acute lesion (Figure 1). The initial MRI mirrored these findings, and DWI did not show any diffusion restriction to suggest an acute territorial infarct (Figure 2).

**Figure 3:**
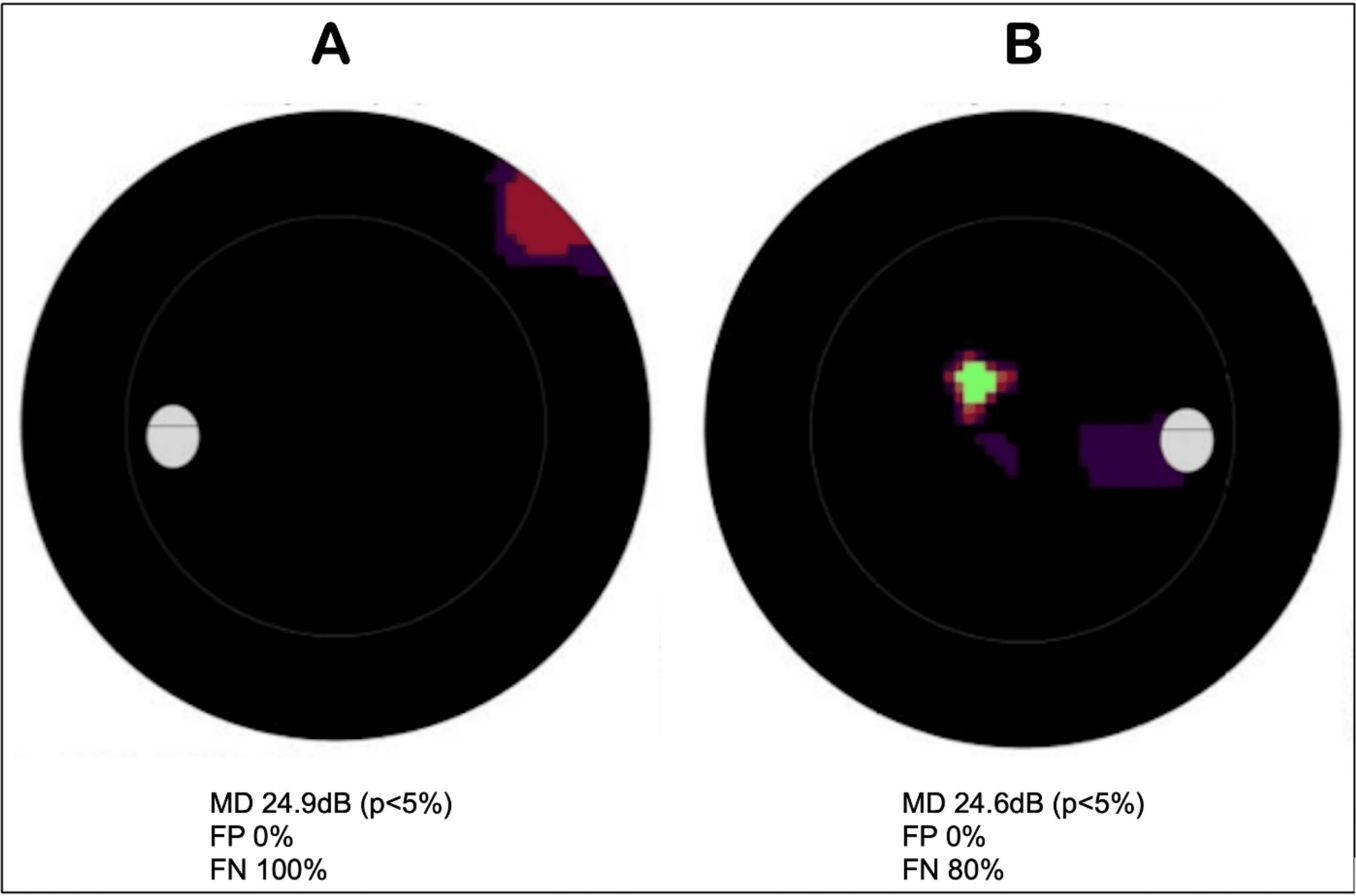
Visual field automated perimetry (Octopus 900 perimeter). Findings shown: Significantly constricted bilateral visual field loss in A) left eye and B) right eye on a greyscale plot. Darker shading indicates areas of decreased vision with high FN values indicating unreliable fields. Normal MD on the Octopus 900 is between 0 and 2dB. MD: mean defect, p: probability value, dB: decibel, FP: false positive, FN: false negative

Shortly after, the patient was admitted to the neurology ward for further evaluation, where her symptoms and signs seemed to fluctuate significantly. The patient reported complete visual loss on admission and was reviewed by the ophthalmology team. Visual field perimetry revealed a markedly constricted bilateral visual field loss (Figure 3) and best corrected visual acuity to perception of light (PL) on the left and 6/48 on the right. Colour vision was 14/19 bilaterally using Ishihara plates, while optical coherence tomography (OCT) showed bilaterally healthy optic discs. There was an incongruity noted between observed behaviour and visual navigation in the patient’s environment and the poor visual assessments, suggesting potential functional visual loss or overlay. An MRI with contrast was organised and was similarly unremarkable. No DWI images were obtained at this time.

Within the second week of admission, a working diagnosis of chronic posterior circulation stroke with functional overlay was given. Visual evoked potentials (VEP) and a bedside cognitive assessment were organised to assess the electrical response of the visual pathways. Unfortunately, these could not be completed due to the patient developing confusion. A standard delirium work-up was completed, including routine blood tests, metabolic and infectious screens, none of which identified a clear cause. Other investigations, including autoimmune panels, paraneoplastic antibody screen, treponemal antibody, and ammonia, were unremarkable (Table 1).

**Table 1:** Laboratory investigations. TSH: thyroid-stimulating hormone, T4: thyroxine, ALP: alkaline phosphatase, ALT: alanine aminotransferase, ESR: erythrocyte sedimentation rate, Ig: immunoglobulin, CTD: connective tissue disease, AMPA: alpha-amino-3-hydroxy-5-methyl-4-isoxazolepropionic acid, CRMP: collapsin response mediator protein 5, K: potassium, GAD: glutamic acid decarboxylase, GABA: gamma-aminobutyric acid, HIV: human immunodeficiency virus

Investigation	Patient value	Reference range
Endocrinology	-	-
TSH	4.2	0.35-5.50 mU/L
Free T4	17.1	10.0-20.0 pmol/L
Cortisol	358	145-619 nmol/L
Biochemistry	-	-
ALP	75	30-130 U/L
Total bilirubin	7	0-20 μmol/L
ALT	19	7-40 U/L
Ammonia	19	12-32 μmol/L
B12	441	211-911 ng/L
Ferritin	34	10-291 μg/L
Folate	11.7	>5.38 μg/L
Homocysteine	12	0.0-16.0 μmol/L
C-reactive protein	5.6	<10 mg/L
Haematology	-	-
ESR	8	5-15 mm/hr
Immunology	-	-
IgG	7.71	6.5-16 g/L
IgA	2.01	0.40-3.50 g/L
IgM	0.35	0.5-3.0 g/L
Bioplex CTD screen	Negative	-
Anti-Yo	Negative	-
Anti-Ri	Negative	-
Anti-Ma1/Ma2	Negative	-
Anti-Hu	Negative	-
Anti AMPA1/AMPA2	Negative	-
Anti-amphiphysin	Negative	-
Anti-CV2/CRMP5	Negative	-
K channel antibodies	Negative	-
Anti GAD	Negative	-
Anti GABA A/GABA B receptor antibodies	Negative	-
Serology	-	-
HIV-1/HIV-2	Not detected	-
Treponemal antibody	Not detected	-

**Figure 4:**
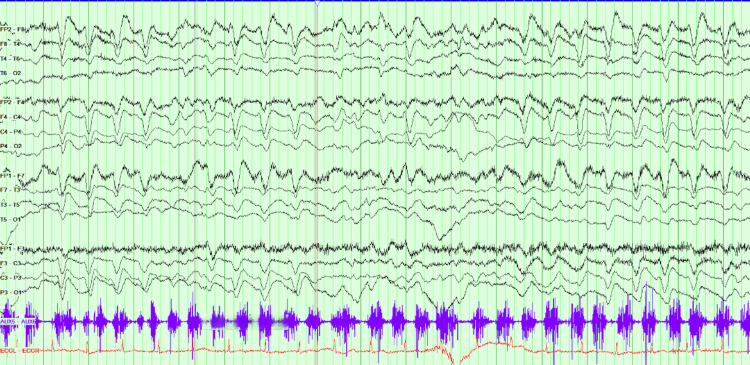
Electroencephalography (EEG). Findings shown: Diffuse background slowing with high-amplitude delta activity of sharpened morphology, maximally frontally, intermittently demonstrating a triphasic pattern. Periods of attenuation to lower-amplitude faster rhythmic activity, with an underlying theta-delta background, consistent with diffuse encephalopathy.

During the third week, the patient became increasingly confused, and prolonged twitching of her left hand had been noted by the nursing staff. The patient was less communicative, with staring spells and reduced movement on the left with semi-purposeful movements on the right, following only a few basic commands. Neurological examination was limited due to the patient's fluctuating level of consciousness. An EEG was performed for suspected non-convulsive status epilepticus, which revealed features of significant diffuse encephalopathy with triphasic appearing discharges (Figure 4), in addition to abnormal asynchronous twitching without an obvious EEG correlate.

**Figure 5:**
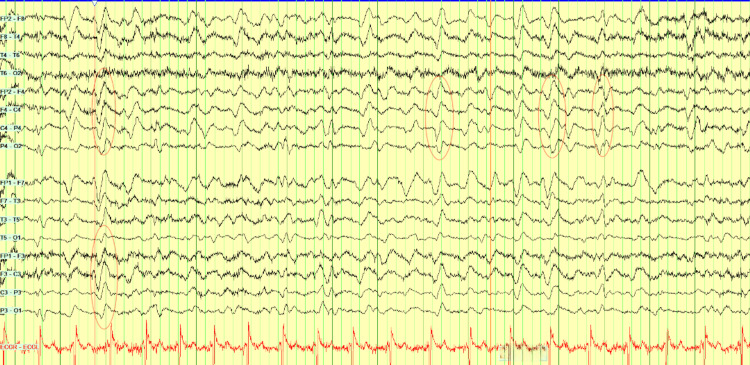
Prolonged electroencephalography (EEG). Findings shown: Markedly slowed background with widespread delta activity. Abundant right-lateralised and generalised periodic discharges with triphasic morphology are present, occurring at up to ~1.5 Hz and demonstrating slight posterior lag. Concurrent limb movements during the recording showed no electrographic correlate.

**Figure 6:**
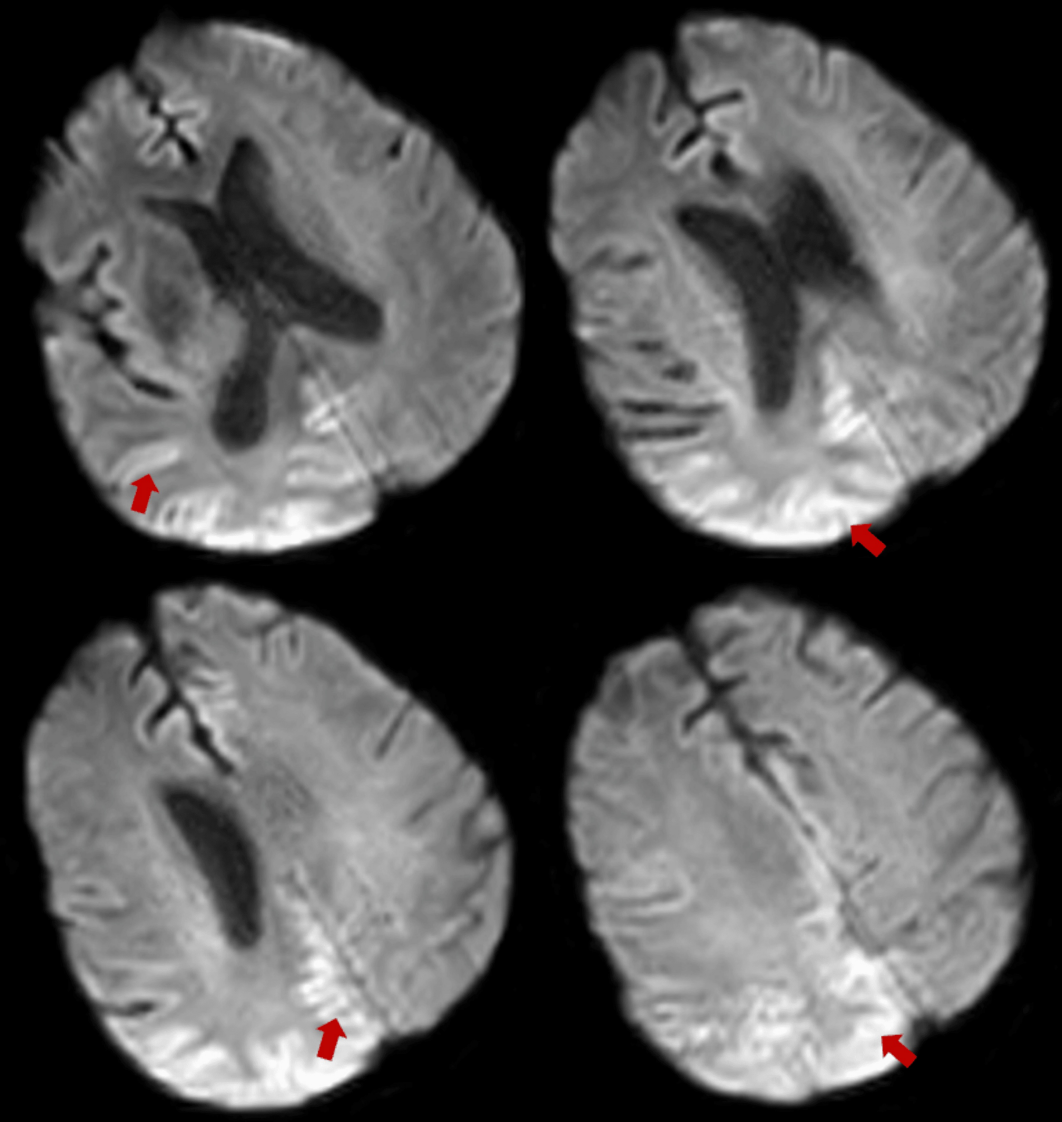
Final MRI brain without contrast. Findings shown: DWI axial sequences showing parietooccipital cortical ribboning highlighted by the arrows. There was significant movement artefact during the study.

A few days later, the patient became unresponsive with worsening orofacial and limb automatisms. A further prolonged EEG was requested, which demonstrated diffuse right lateralised and generalised periodic sharp wave complexes with a triphasic morphology suggestive of prion disease (Figure 5). Repeat MRI showed diffusion restriction within the right parietal and occipital cortices, as well as within the cingulate gyrus (Figure 6). A lumbar puncture was not performed due to the patient’s advanced state, and further invasive testing was deemed unlikely to add diagnostic value. The patient's overall clinical presentation, EEG, and MRI findings were sufficient to make a diagnosis of probable Heidenhain-variant sporadic CJD. She was transitioned to palliative care, discharged to a hospice, and died a few days after, almost six weeks from the day of admission.

## Discussion

Although patients may develop visual symptoms at some point during the progression of CJD [6], the isolated visual symptoms at the onset of presentation with preservation of cognition are characteristic of HvCJD [7]. These visual symptoms include visual field defects, astereopsis, double vision, visual loss, and dyschromatopsia [6,8]. The most common cause of homonymous hemianopia in adults is an ischemic stroke affecting the occipital cortex [9]. Ataxia and diplopia are common findings of posterior circulation stroke, with a hyperacute or space-occupying lesion if more of an insidious onset. However, this is usually supported with relevant findings on brain imaging.

Other differentials that should be considered include demyelination/inflammation, paraneoplastic syndromes, toxic/metabolic, vasculitis, occipital lobe seizures, etc. In view of her age and progressive unexplained visual changes, posterior cortical atrophy (PCA) secondary to Alzheimer's disease or corticobasal degeneration is also a consideration (Table 2). Functional visual loss is diagnosed based on positive clinical features supporting normal vision rather than by exclusion [10]. The absence of identifiable organic pathology alone should not be taken as sufficient evidence for a functional diagnosis. One way to confirm functional vision loss is the use of an optokinetic drum, which elicits a reflexive optokinetic nystagmus [10] as the patient’s eyes involuntarily track its black and white stripes. The diagnosis of a functional disorder in this case was supported by variability in visual examinations, normal initial investigations, and incongruity with observed interactions with their surroundings. However, progression of the patient’s condition to akinetic mutism was inconsistent with a functional disorder, warranting repeat investigations, which eventually led to a diagnosis of HvCJD.

**Table 2:** Differential diagnoses for acute/subacute visual field loss and ataxia. Summary of other diagnoses considered during the evaluation of this case and reasons for exclusion. AD: Alzheimer's disease, CBD: corticobasal degeneration, TIA: transient ischemic attack, MS: multiple sclerosis, ADEM: acute disseminated encephalomyelitis, CNS: central nervous system, GCA: giant cell arteritis, CT TAP: CT of the thorax, abdomen, and pelvis, MELAS: mitochondrial encephalomyopathy, lactic acidosis, and stroke-like episodes, MIDD: maternally inherited diabetes and deafness

Differential	Supporting Features	Reasons for exclusion in this case
Posterior Cortical Atrophy (PCA) due to AD or CBD	Gradual visual dysfunction, visuospatial deficits; cognitive decline; posterior atrophy on MRI	Rapid progression and encephalopathy inconsistent with PCA
Vascular (Stroke/TIA)	Sudden onset of homonymous hemianopia and ataxia suggests posterior circulation involvement [9]; history of previous posterior circulation stroke	Initial CT and MRI were normal; no vascular lesion identified
Demyelination (e.g. MS, ADEM)	Can present with visual field loss and ataxia [11]; presence of demyelinating lesions on MRI; often relapsing-remitting pattern	Unlikely due to age, lack of prior episodes, and absence of demyelinating MRI features
Progressive Multifocal Leukoencephalopathy (PML)	Subacute visual symptoms due to occipital white matter involvement; associated with immunosuppression [11]	No evidence of immunosuppression; viral screen was negative
Paraneoplastic Syndromes	Vision loss and ataxia can be present; antibody-mediated [11]	CT TAP not done; however, no positive antibodies (anti hu, yo, ri, crmp5/cv2, ma1/ma2 negative)
Toxic/Metabolic Encephalopathy	Visual or cerebellar disturbance may be present; May be due to hepatic, mitochondrial, or hypoglycaemic causes [11]	Normal metabolic and hepatic profiles; postmortem mitochondrial genetic testing for MELAS and MIDD were negative
Vasculitis (e.g., CNS vasculitis, GCA)	May cause multifocal ischemia or visual loss; elevated ESR/CRP	Normal inflammatory markers and imaging; no systemic features
Occipital Lobe Seizures	Brief (<2 mins) visual phenomena or field defects, temporary blindness; EEG changes	Persistent and progressive symptoms; absence of epileptiform activity on EEG

While MRI is an important tool for antemortem diagnosis of CJD with high specificity and sensitivity, particularly DWI sequences with correlating ADC map, its limitations are evident in this case. The diagnostic criteria for probable CJD include the presence of hyperintensities either in the basal ganglia or at least two cortical regions (parietal, temporal, or occipital) on FLAIR or DWI sequences [12]. Early in the disease course, typical MRI abnormalities may be subtle, missed, or even absent [13], as seen in some sCJD subtypes. EEG remains an integral diagnostic tool, but similar to imaging, early changes may be non-specific. Changes exhibited depend on the stage of disease: slowing in early disease to periodic sharp wave complexes (PSWCs) in middle-to-late stages, and coma traces just before death [14]. In a retrospective study of 20 patients with HvCJD, Yang et al. reported that characteristic MRI and EEG abnormalities were not identified on initial investigations in a substantial proportion of cases, with MRI DWI changes recognised at a median of six weeks after reported symptom onset and PSWCs on EEG at a median of eight weeks [15]. Perhaps, these findings reflect the subtlety and potential under-recognition of early abnormalities rather than the true absence of pathology, particularly in visual-onset disease. Therefore, serial imaging and EEG should be carried out if clinical features raise high suspicion for CJD.

CSF analysis is another key investigation for the diagnosis of sCJD. RT-QuIC can detect the presence of misfolded prion protein (PrPSc) even in small quantities, with sensitivity and specificity of >90% and 100%, respectively [16]. A CSF assay positive for 14-3-3 or tau proteins can also support the diagnosis of HvCJD. The sensitivity of 14-3-3 protein is 86%, but as a marker of neuronal damage, it can be raised in other neurological conditions [12,16]. CSF t-tau protein has a sensitivity and specificity of 91.3% and 78.7%, making it a more reliable biomarker than 14-3-3 [15]. Nevertheless, RT-QuIC remains the most accurate tool in sCJD diagnosis, with the second-generation assay (IQ-CSF) having a lower threshold for detecting prion seeding, increasing sensitivity to 97.2% [17]. Early CSF RT-QuIC assay is advised when there is no clear cause for visual loss with or without rapid neurological decline. However, this must be interpreted with caution as RT-QuIC can be negative in slower progressing subtypes and in younger age of onset [17].

Limitations of this paper include being a single case, restricting the application of its findings to a broader population. A lumbar puncture was not performed for diagnosis, and a post-mortem examination was not undertaken; therefore, a definitive diagnosis of prion disease was not made.

## Conclusions

This case demonstrates how HvCJD can mimic a stroke or functional visual loss at onset with normal early investigations. The fluctuating nature of symptoms and preserved visual pathways on imaging can resemble features of a functional disorder, yet the subsequent rapid progression to confusion and akinetic mutism is characteristic of an evolving organic encephalopathy. A single negative MRI does not exclude CJD; repeat MRI and EEG should be considered, as characteristic diagnostic features may become evident only later in the disease course. Importantly, early lumbar puncture should be considered in these patients to prevent diagnostic delay and guide timely palliative care.
